# An overview of a different type of cardio-oncology gathering: summary of the COMP (cardio-oncology multidisciplinary practice) meeting held in Houston Texas, January 2020

**DOI:** 10.1186/s40959-020-00075-7

**Published:** 2020-09-15

**Authors:** Anecita P. Fadol, Nicolas L. Palaskas, Michael S. Ewer, Anita Deswal

**Affiliations:** 1 Department of Nursing, University of Texas MD Anderson Cancer Center, 1515 Holcombe Blvd., Houston, TX 77030-4009 USA; 2 Department of Cardiology, University of Texas MD Anderson Cancer Center, 1515 Holcombe Blvd., Houston, TX 77030-4009 USA

**Keywords:** Cardio-oncology, Conference, Multidisciplinary

## Abstract

An innovative Cardio-Oncology meeting was held in Houston, Texas in January of 2020. This gathering was intended to broaden the scope of Cardio-Oncology to include major presentations by clinicians and researchers beyond physicians, as well as to provide comprehensive reviews by established experts aimed at a variety of levels of professional practitioners. The unique perspective of this meeting is presented in the overview that follows. This overview is intended to contribute to a broader view of Cardio-Oncology, and to provide perspective for the expanding group of providers relative to their individual areas of expertise. These perspectives can and should be incorporated in cardio-oncology centers.

## Background

The close association between cardiac abnormalities and cancer is increasingly appreciated. It stems from several considerations: some obvious, others much subtler. Among the obvious is that much of cancer is, as is heart disease, more prevalent in older populations. Additionally, there are common risk factors that may give rise to both malignant and cardiac disease. Were these the only relevant considerations, the broad interest in the interaction between the disciplines of cardiology and oncology would have been less likely to have evolved over the past half century as it has. It is the less obvious considerations that allow for the meaningful interventions that have spurred interest in cardio-oncology; among the more important, the observation that for many of the anti-cancer drugs that impede cell growth but at the same time destroy myocardial contractile elements or cells, do so through different mechanisms, thereby allowing the desired anti-tumor effect to be selectively enhanced while maintaining an acceptable level of cardiac injury. As newer agents enter the clinical armamentarium of the oncologist it becomes increasingly apparent that these agents compromise cardiac function in different ways and through different mechanisms; not all cardiotoxicity is created equally. Interestingly, vascular abnormalities were among the first entities to be recognized and that would ultimately fit into the realm of cardio-oncology, but our understanding of both tumor effects and the unintended sequalae of anti-cancer treatment on the vasculature has offered a new dimension to this evolving discipline. This article reports on and summarizes the highlights of this session.

## Introduction

The inaugural Cardio-Oncology Multidisciplinary Practice (COMP) meeting was held on January 17th and 18th and was co-chaired by Dr. Anecita Fadol and Dr. Nicolas Palaskas. Based on feedback from prior internal educational sessions and the results of the needs assessment survey for oncology nurses, we formed an interdisciplinary program planning committee to address these needs in the conference. The committee was composed of staff nurses, advanced practice nurses (APN), physician assistants, and a cardiologist who all had experience in treating patients with cardiovascular disease and cancer in the inpatient and outpatient setting.

The meeting was hosted by MD Anderson Cancer Center in Houston, Texas. The intent of this conference was to expand the concerns and triumphs of cardio-oncology to a broader group of researchers and practitioners, and to update health care providers using a multidisciplinary approach in the management of cardiovascular issues in patients with cancer across the continuum of care from diagnosis, active treatment, to survivorship or supportive/palliative care at end of life. These interrelated concepts provided the attendees with essential knowledge for starting a cardio-oncology program or expanding their already existing cardio-oncology practice. In all, over 150 registrants from all over the nation that included nurses, advanced practice providers, and physicians. The organizers wanted to create an environment substantially different from previous meetings and symposia to reflect that this field now encompasses perspectives beyond those of physicians engaged in the practice of cardiology or oncology. Cardio-oncology, to achieve its ultimate goal of optimizing oncologic benefit within the confines of acceptable and minimized cardiac risk in patients both with and without underlying or anticipated cardiac burdens or risk, must appreciate the increasingly important role of practitioners beyond physicians. This conference was focused on the broad experiences of nurse-practitioners, physicians’ assistants, supportive care experts, and ethicists along with updates provided by physicians with focused knowledge in some of the less well-appreciated nuances of Cardio-Oncology, and information regarding these sessions was available widely through the conference services resources within our institution.

**Fig. 1:**
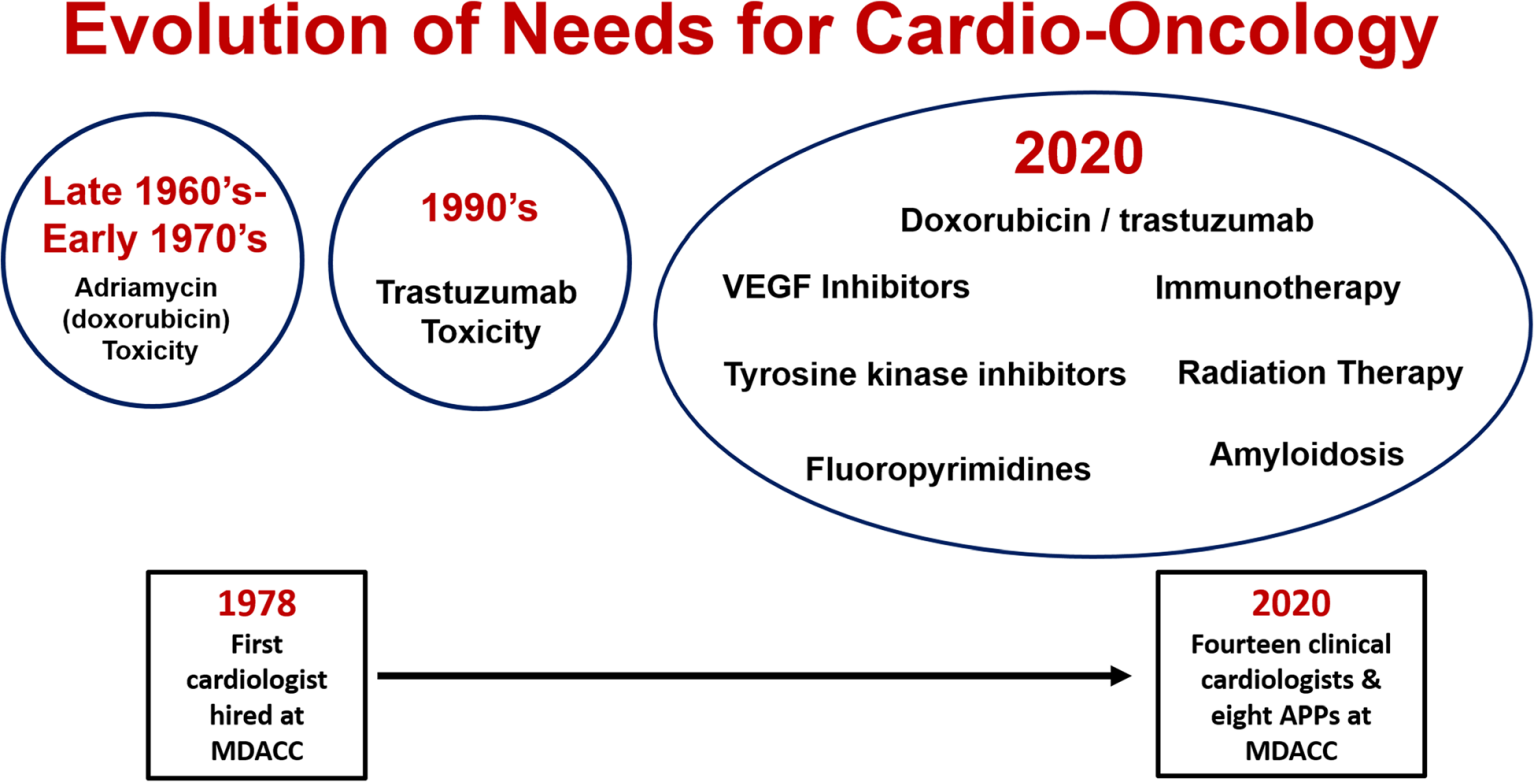
Evolution of Cardio-Oncology and the growth of MD Anderson’s cardiology department presented by Dr. Anita Deswal

It was in this vein that Dr. Anita Deswal and Dr. Carol Porter provided opening remarks to set the stage for the scientific and clinical presentations. Dr. Deswal opened with an overview of the public health implications for cardio-oncology, and the shifting balance between cardiac disease that constitutes a major risk factor for subsequent cancer interventions, and the effects of these interventions on the heart (Fig. 1). Dr. Porter went beyond considerations of the diseases themselves to emphasize how important a humanistic approach is to improve the overall quality of life of patients affected by both cancer and heart disease.

### Cancer therapeutic cardiovascular side effects

The first of the scientific sessions, moderated by Joanne Dalusung, advanced practice nurse (APN), incorporated three presentations by pharmacists; the first by Dr. Ali Zalpour, discussed the various cardiovascular sequelae of newer cancer therapeutics, emphasizing concerns beyond left ventricular function to include arrhythmias, thrombosis, pulmonary hypertension, and coronary artery disease. Prolongation of the Q-T interval has received considerable attention; the true impact on the interval by any particular agent is often challenging to determine due to compounding factors such as polypharmacy and electrolyte abnormalities. The importance of drug interactions, despite considerable attention, is often under-appreciated by clinicians. Newer cancer treatments may augment the cancer-related tendency to interfere with or augment the clotting cascade, and thromboembolism, both venous and arterial, with ischemic considerations ensue. Unusual presentations may occur and at times require urgent intervention. Dr. Justine Wang looked at some of the mechanisms of cardiotoxicity and risk factors related to trastuzumab-induced myocardial dysfunction and the ischemic phenomenon related to the fluoropyrimidines. Various guidelines, including the American Society of Clinical Oncology (ASCO) clinical practice guidelines, regarding management strategies were discussed. Dr. Maggie Lu focused on anthracycline cardiotoxicity discussing the generally accepted and widely disseminated concepts regarding risk factors and monitoring considerations, as were the long-term implications of treatment in children.

### Cardiovascular toxicity with immunotherapy

**Fig. 2:**
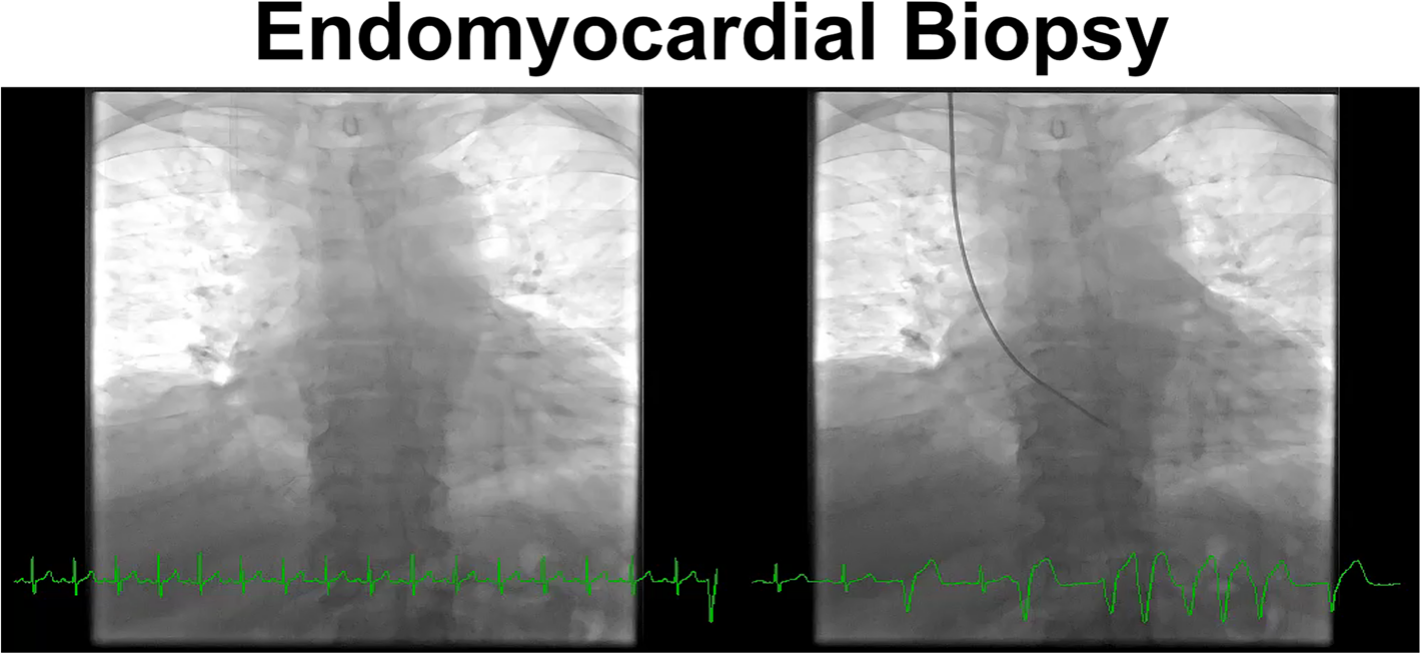
Endomyocardial biopsy for the diagnosis of immune checkpoint inhibitor associated myocarditis as presented by Dr. Nicolas Palaskas (previously unpublished image)

Dr. Nicolas Palaskas reviewed the current understanding of cardiac sequelae related to modern immunotherapies focusing on chimeric antigen receptor T cell (CAR-T) therapy, cytokine release syndrome and immune checkpoint inhibitor (ICI) associated myocarditis. CAR-T therapy is mostly associated with tachyarrhythmias secondary to cytokine release and ultimately treated with suppressing the cytokines and supportive management. He emphasized that although ICI associated myocarditis is reported to be uncommon it is likely under-appreciated and carries a high mortality. Some non-specific symptoms such as fatigue and dyspnea may be harbingers of more serious inflammation that may be difficult to detect using a single cardiac test. Multimodality imaging in combination with invasive techniques such as endomyocardial biopsy are required to diagnose and treat ICI myocarditis more appropriately. Findings that may be seen on electrocardiograms include prolongation of the PR interval, advanced atrioventricular block, and ventricular tachycardia. Early during the course of this troubling entity, left ventricular systolic function often remains normal despite patients presenting with florid heart failure and therefore a strong clinical suspicion should prevail. Magnetic Resonance Imaging may identify related edema and necrosis/fibrosis; pericarditis and pericardial effusion may also provide supportive diagnostic value. Dr. Palaskas reviewed the technique of endomyocardial biopsy and the resurgence of this invasive procedure in the context of diagnosing myocarditis (Fig. 2). The various therapies, with their present perceived likelihood of positively impacting the disease process was discussed.

### Overview of cardiovascular testing

Ms. Saleemah Smith, physician assistant (PA-C) reviewed the basic and more complex testing modalities used by the Cardio-Oncology team. She emphasized that while their implementation may be clear to the ordering clinician, patient education is essential to ensure understanding of the implications of testing results on future interventions and cancer therapy. Ms. Smith outlined the indications and limitations of our non-invasive tests along with the rare but important adverse events that may occur with the more invasive techniques. Ms. Smith stressed the huge importance to our patients of proper explanations regarding these tests, especially in an era where invasive interventions are becoming more common.

### Preoperative risk stratification prior to oncologic surgery

Ms. Natascha Rivas, PA-C and Dr. Jose Banchs emphasized the importance of individualized assessment in estimating the cardiac risks of non-cardiac interventions for the treatment of cancer. The balance of underlying cardiac risk with the anticipated stress of the intervention, be they surgical or non-surgical modalities is of fundamental importance and easily overlooked. Among the less well-appreciated aspects of these evaluations is the need to modify standard algorithms and recommendations for stented patients requiring interruption in their anti-coagulation, and updated considerations for patients with chemotherapy-induced thrombocytopenia. Some of the pitfalls regarding interpretation and estimation of cardiac systolic function were discussed, as was the benefits of echocardiographic contrast agent image enhancement, three-dimensional imaging, and deformation patterns (strain imaging)—modalities gaining support and recognition in the Cardio-Oncology practice. The selection of imaging technique, the need for quantification methods, newer modalities such a three-dimensional imaging, contrast enhancement, and speckle tracking (strain) should enjoy more widespread use as clinicians gain familiarity with these techniques.

### Complexities of the patient with Cancer

The final presentation of the morning session was presented by Ms. Myrshia Woods, PA-C who provided a global perspective of patient care for the complex patient seen in a Cardio-Oncology setting. Inevitably, the cancer patient endures various side effects including extreme nausea/vomiting, mucositis, malnutrition, anemia, electrolyte abnormalities and fatigue, and these factors undoubtedly impact their cardiovascular health in the form of volume overload/contraction, hypertensive emergencies, arrhythmias, and heart failure. Due to these side effects patients may not be able to take their medications during these times, furthering additional metabolic compromise that may be life-threatening. It was stressed that providing comprehensive education and emotional support to both patients and family members/caregivers helps to improve patient self-monitoring, timely reporting of cardiac symptoms, and implementation of heart healthy lifestyle practices. This will lead to improved cardiac medication compliance as well as better cardiovascular health and survival for the cancer survivor.

### Anticoagulation considerations in Cancer patients

Afternoon sessions were kicked off with Ms. Hasana O’Neal Herbert, PA-C discussing the use of anticoagulants for deep venous thrombosis, pulmonary embolism, and stroke risk mitigation in atrial fibrillation. Special attention was made for consideration of thrombocytopenia and the MD Anderson protocols for holding and restarting anticoagulation in the setting of rapidly falling or chronic thrombocytopenia. Various risk scores including the CHADS2VASc and HASBLED scores were presented in terms of their applicability to cancer patients and lack of certain parameters such as intraluminal gastrointestinal malignancies and intracranial metastases that may increase a patient’s risk of bleeding complications. The limited trial evidence for the use of direct oral anticoagulants (DOACs) compared to heparin and warfarin in cancer patients was presented but contrasted by the real-world experience of increasing adoption of DOAC use in MD Anderson’s daily clinical practice.

### Electrocardiogram monitoring during chemotherapy

Dr. Peter Kim, director of electrocardiogram and telemetry monitoring at MD Anderson, discussed the management of arrhythmias encountered in cancer patients and the importance of QT prolongation monitoring in both clinical practice and clinical trial drug monitoring. The management of arrhythmias focused on common arrhythmias encountered during inpatient admissions such as atrial fibrillation with rapid ventricular rate, atrioventricular block, and torsades de pointes. Importance was placed on considering drug-drug interactions when choosing antiarrhythmic medications. The benefits and indications for the use of telemetry monitoring were emphasized highlighting situations in which continuous monitoring demonstrates benefit or not.

### Implantable cardiac devices in Cancer patients

Dr. Kaveh Karimzad, electrophysiologist at MD Anderson, gave an overview of implantable pacemakers, defibrillators, and loop recorders. The indications for pacemaker implantation and cardiac resynchronization therapy (CRT) were reviewed. Dr. Karimzad presented that limited evidence exists that shows similar benefit of CRT in patients with cancer therapeutic induced cardiac dysfunction as is seen in heart failure from other causes. The unique challenges and complications that can arise in patients needing implantable devices were presented with cases describing the issues with neutropenia and bacteremia in cancer patients with devices leading to endocarditis and thrombocytopenia leading to special measures to limit periprocedural bleeding.

### Cardiac catheterization laboratory in a Cancer hospital

Along the theme of invasive procedures, the director of the cardiac catheterization laboratory Dr. Cezar Iliescu and the catheterization lab manager Gerry Tomakin, MSN presented the MD Anderson experience of implementing the first cardiac catheterization laboratory in a primary cancer center. The catheterization laboratory performs various procedures including coronary angiography, left and right heart catheterization, pericardiocentesis, endomyocardial biopsy, and implantable cardiac devices (pacemakers, defibrillators, loop recorders). In addition, advanced diagnostics are performed including fractional flow reserve, intravascular ultrasound, and optical coherence tomography. Cancer patient complexities including older age, frailty, multiple comorbidities, coagulopathies, and immunosuppression make a team-based approach to caring for these patients essential to limit complications. These complexities are managed by performing extra precautions such as performing surgical prep twice in neutropenic patients, using micro-puncture kits and ultrasound for all vascular access, and use of bi-plane imaging to reduce contrast exposure.

### Carcinoid heart disease

Dr. Saamir Hassan gave a comprehensive lecture regarding the carcinoid heart disease including the pathophysiology, clinical presentation, diagnosis, and treatment. This is a rare disease but as a tertiary referral center, MD Anderson has a significant cohort of carcinoid heart disease patients who are co-managed between cardiology, gastrointestinal medical oncology, and cardiothoracic surgery collaborators specializing in tricuspid and pulmonic valve replacement at the University of Texas Memorial Hermann Medical Center in Houston, Texas. The mechanistic pathways of how carcinoid tumor releasing serotonin leads to valve destruction were displayed. The typical echocardiographic imaging findings of carcinoid heart disease were presented including tricuspid and pulmonic valve leaflet restricted motion and regurgitation. The available evidence regarding timing of valve surgery, choice of prosthetic valves, and perioperative anesthesia management to avoid carcinoid storm were presented.

### Trastuzumab Cardiotoxicity

**Fig. 3:**
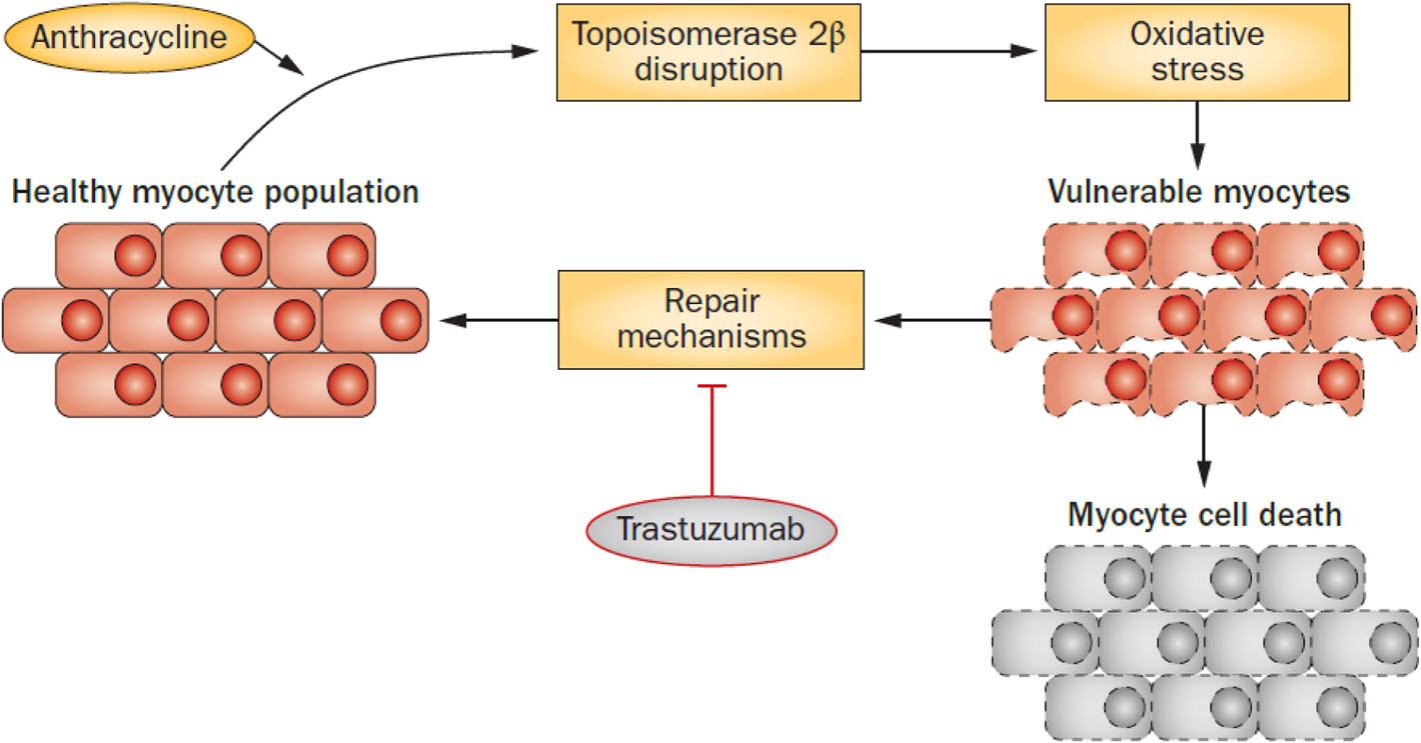
A proposed mechanism for the anthracycline-trastuzumab interaction. The repair of vulnerable myocytes is impeded by trastuzumab, furthering the pathway toward cell death [1]

Dr. Michael S. Ewer, delivered a thought-provoking speech regarding the history of trastuzumab cardiotoxicity. The caveats of asymptomatic versus symptomatic declines in left ventricular ejection fraction, variability in cardiac imaging results, and potential mechanisms of toxicity were discussed. He presented the differences in toxicity of doxorubicin versus trastuzumab and how the two given concomitantly may have led to initial reports of high toxicity. Doxorubicin causes direct myocyte damage seen on endomyocardial biopsy even at low doses but trastuzumab has never been shown to have distinct changes on endomyocardial biopsy. This leads to the thought that trastuzumab affects myocardial repair mechanisms therefore its toxicity is only clinically apparent when the heart is also being damaged by a direct cardiotoxin (Fig. 3).

### Pericardial disease in Cancer patients

**Fig. 4:**
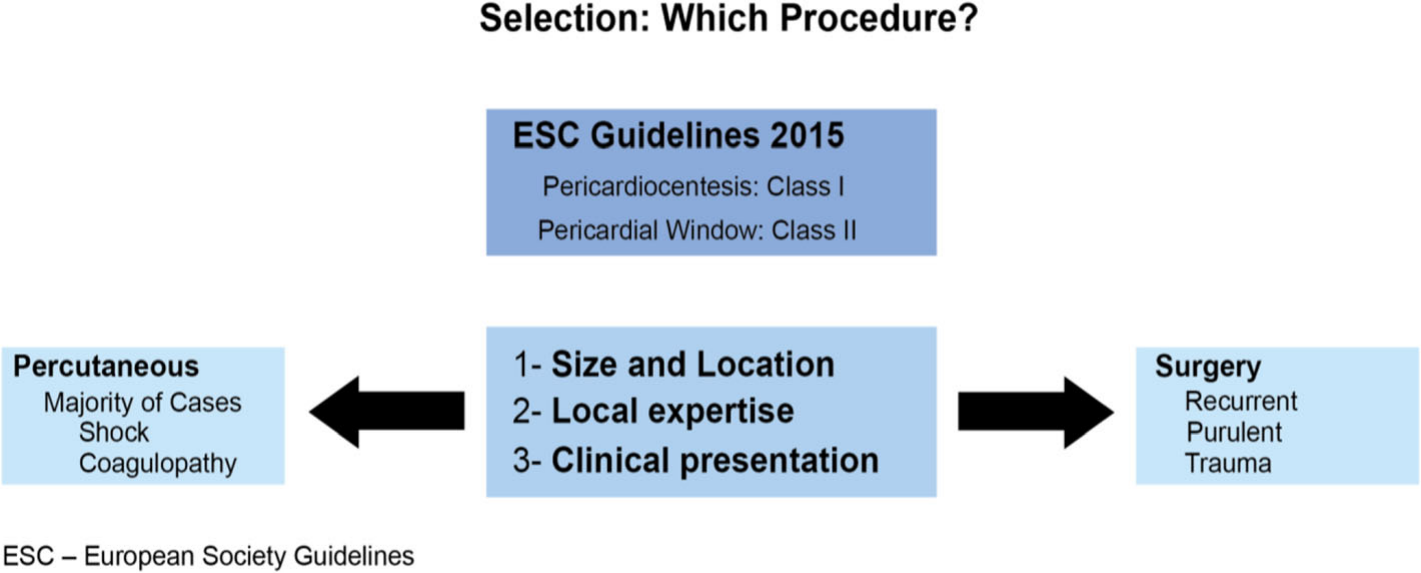
Algorithm for relief of pericardial effusion in Cancer Patients [2]

The diagnosis and management of pericardial disease in cancer patients was presented by Dr. Elie Mouhayar. Focusing on pericarditis, pericardial effusion, and constrictive pericarditis, Dr. Mouhayar presented how several different cancer therapeutics including radiation may lead to these cardiotoxicities. Various diagnostic imaging modalities were discussed such as electrocardiograms, echocardiograms, and computed tomography. Considerations of when to perform a percutaneous pericardiocentesis and how to select the appropriate procedure were highlighted (Fig. 4). An algorithm using both non-invasive tests, echocardiography and cardiac magnetic resonance imaging, and invasive left and right heart catheterization for diagnosing constriction were also presented.

### Radiation-induced heart disease

**Fig. 5:**
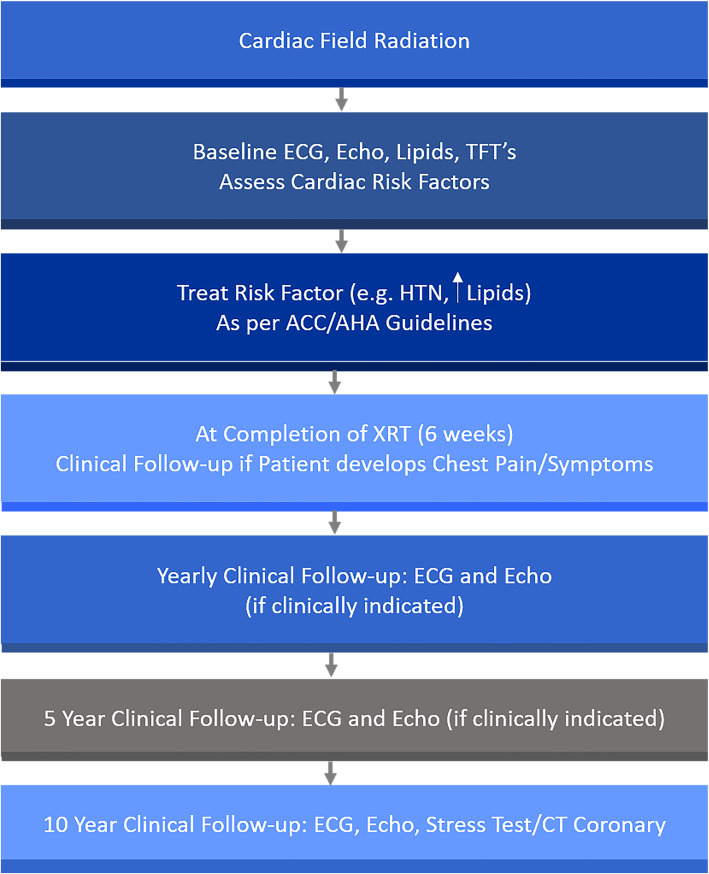
ACC/AHA=American College of Cardiology/American Heart Association; CT=Computerized Tomography; ECG=Electrocardiogram; Echo=Echocardiogram; HTN=Hypertension; TFT=Thyroid function tests; XRT=Radiation therapy [3]

The day was concluded with a talk from Dr. Wamique Yusuf discussing radiation-induced heart disease (RIHD). The risk factors for developing clinically relevant heart disease from radiation are age, time from radiation, traditional cardiac risk factors, and > 30 Gy dose of radiation were presented. The cumulative incidence of heart disease increases as time increases from the time of receiving radiation when compared to matched controls. Several different clinical manifestations of RIHD are possible; including endothelial dysfunction, coronary artery disease, pericardial disease, valvular disease, conduction system disease, and heart failure from myocardial fibrosis. The long-term follow algorithm for patients that received radiation was also presented (Fig. 5).

### Cardiac amyloidosis- keys for diagnosis

**Fig. 6:**
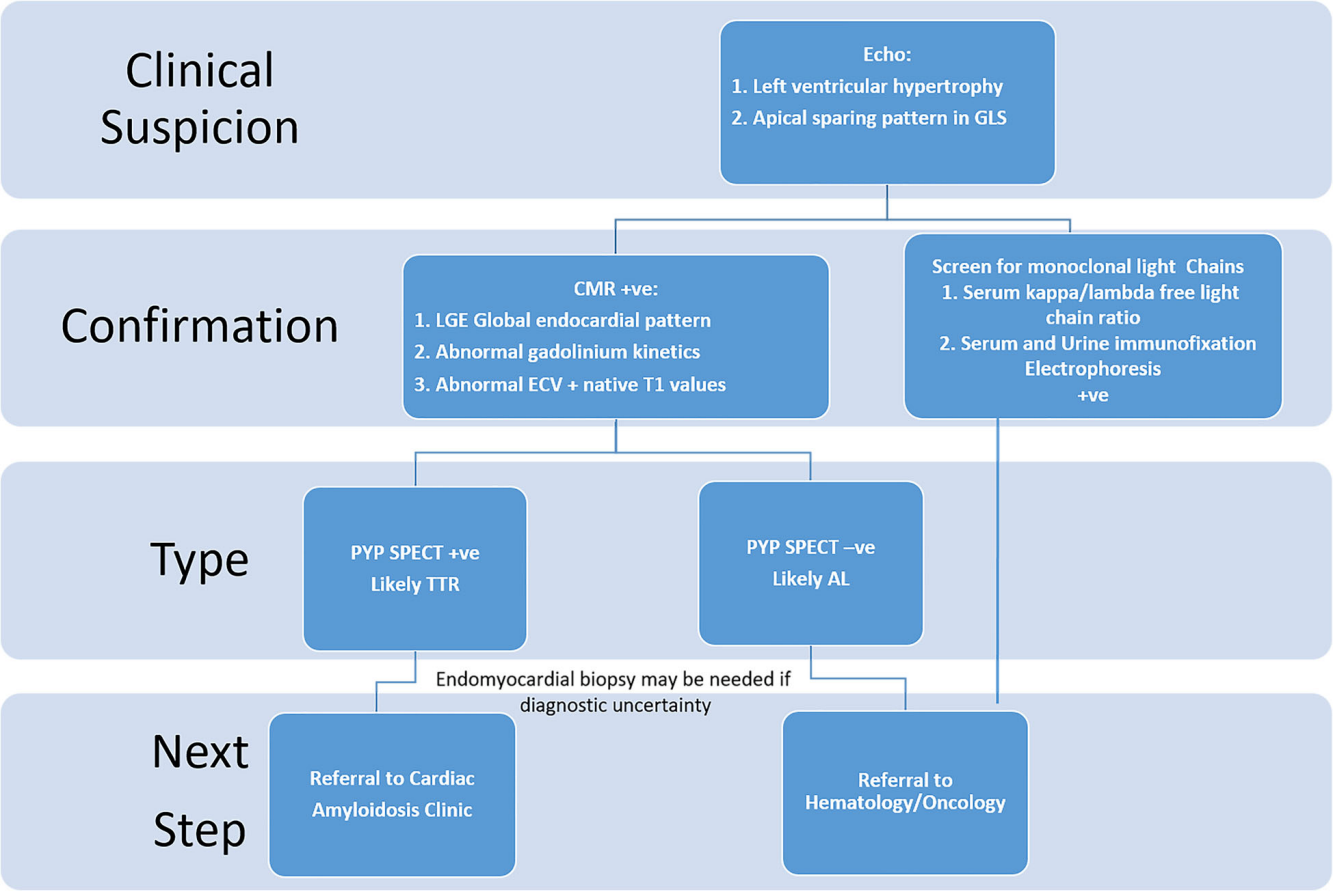
Diagnostic algorithm for workup of clinically suspected cardiac amyloidosis. Modified from Agha AM, et al. Role of cardiovascular imaging for the diagnosis and prognosis of cardiac amyloidosis. Open Heart. 2018 Sep 26;5 (2):e000881.doi:10.1136/openhrt-2018-000881. eCollection 2018. review. AL,light chain amyloidosis; ATTR,abnormal transthyretin; CMR, cardiovascular magnetic resonance; DPD, 3,3-diphosphono-1,2-proporanodicarboxylic acid; ECV, extracellular volume; GLS, global longitudinal strain; LGE, late gadolinium enhancement; PYP, pyrophosphate; SPECT, single photon emission CT [4]

The second day of the conference was initiated by Dr. Juan Lopez-Mattei who discussed the diagnosis and management of amyloidosis, both transthyretin (TTR) and light chain (AL). The differences in clinical presentation including macroglossia, carpal tunnel syndrome, and periorbital ecchymoses were presented including diagnostic algorithms to differentiate the two types of amyloid. The treatments for TTR and AL amyloidosis are very different, therefore it is important to accurately diagnose which subtype is present. In addition, the early mortality of AL amyloidosis is high, therefore treatment needs to be initiated as soon as possible. Echocardiography can show increased wall thickness and apical sparing pattern on the global longitudinal strain polar map, but is not a good test to differentiate between AL and TTR. Nuclear imaging with pyrophosphate tracer (PYP) will show increased myocardial uptake with TTR and not with AL and can help to determine if one has TTR or not. Cardiac magnetic resonance imaging typically shows a sub endocardial pattern of late gadolinium uptake and its presence is related with bad prognosis. In combination with serum free light chain testing, the imaging diagnostic algorithm was discussed (Fig. 6).

### Imaging of Intracardiac tumors

Dr. Kara Thompson presented on cardiac tumors and how to diagnose them with various imaging modalities. Intracardiac tumors are rare and differentiation between imaging artifact and a true mass is the first distinction to make. The second process is to determine if the mass is a tumor, thrombus, or vegetation. The differences in imaging qualities of tumor versus thrombus were discussed for both echocardiography and cardiac magnetic resonance imaging. After determining the mass is a tumor then fine imaging points to determine if it is a benign versus malignant tumor such vascularity, borders, heterogenous echodensity, and echo contrast uptake were presented. Metastatic disease to the heart is more common than primary cardiac tumors, and benign primary cardiac tumors are more common than malignant primary cardiac tumors. MD Anderson’s multidisciplinary tumor board experience and collaboration with Dr. Michael Reardon, cardiothoracic surgeon specializing in intracardiac tumor removal, was described.

### Cardio-oncology program development

Developing a Cardio-Oncology program is a team sport. Collaboration among the interdisciplinary professionals involved in the management of patients is critical for a successful Cardio-Oncology Program. Dr. Anecita Fadol, nurse practitioner (NP) at MD Anderson and Nadine Henning, NP from Indiana Community Health Network, presented an exemplar of their experiences in developing a successful hospital- based and community-based Cardio-Oncology programs. In both hospital and community settings, they emphasized the importance of a Cardio-Oncology program for the prevention, early recognition and mitigation of the effects of cancer treatments on the cardiovascular system. Given the increasing number of Food and Drug Administration (FDA)-approved anti-cancer agents that have potential cardiotoxic effects; and with cancer and heart disease sharing similar risk factors, improved screening prior to starting anti-cancer therapy is paramount to prevent cardiotoxicity. The challenges for clinicians are to identify which preventive strategies should be used to minimize risk for cardiotoxicity prior to initiation of therapy, during treatment with potentially cardiotoxic anti-cancer agents, and to determine the preferred approaches for surveillance and monitoring after treatment for patients at risk for developing cardiac dysfunction.

**Fig. 7:**
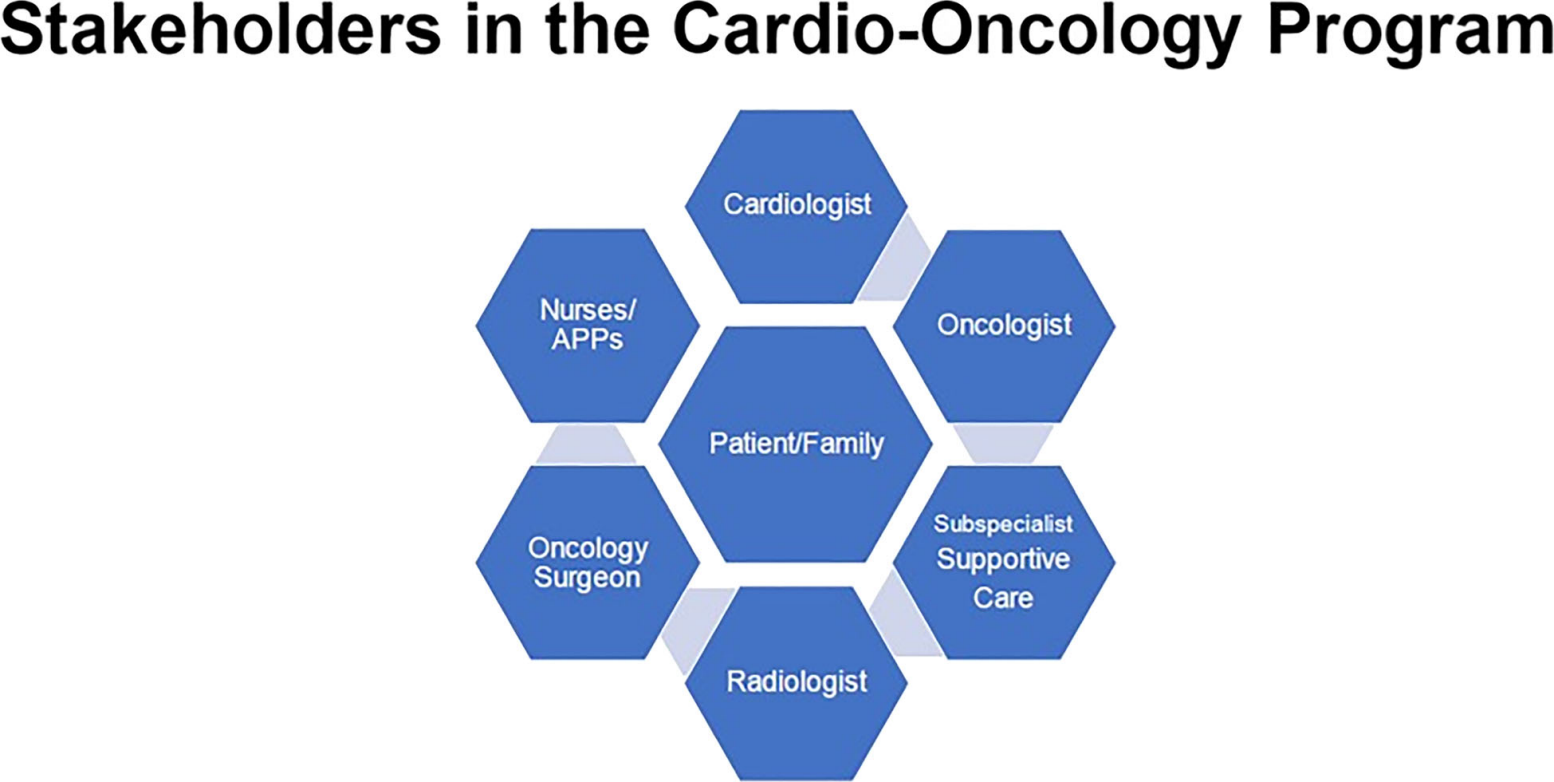
The key components of a cardio-oncology program and the all of the stakeholders involved were presented by Dr. Anecita P. Fadol

The key components for the development of a successful Cardio-Oncology program was also discussed (Fig. 7). In the planning stage of developing a program, it is a must to have a shared leadership between cardiologist and oncologist. In addition, a commitment from hospital or community center administrators’ is critical to provide support of the strategic operations, financial planning and overall management of the program. The location of the clinic, particularly for the community-based clinic, should be in close proximity to the cancer center or the hospital to facilitate patient outpatient follow up visits with other oncology appointments. Access to a cardiovascular testing center with facilities for diagnostic and interventional procedures, such as echocardiography including strain measurement should be considered. Education of the staff, patients and families should be included in the planning.

The role of the nurses and advanced practice providers (APPs) in the inpatient and community-based Cardio-Oncology programs were highlighted, particularly in facilitating direct communications between cardiology and oncology services, follow up of patients in the outpatient clinic, including titration of medications, and triage urgent cardiovascular issues.

Ms. Henning highlighted the strategies to increase referrals to the community-based Cardio-Oncology clinic by publicizing the value of the program including multimedia marketing, use of physician referral cards, patient brochures and networking at professional meetings.

### Cardiovascular issues in Cancer survivorship

Dr. Alma Rodriguez, Director of Cancer Survivorship, discussed cardiovascular issues to consider in cancer survivors. There are three stages of cancer survivorship including the acute, intermediate, and long-term. The importance of risk factor mitigation in cancer survivors was emphasized including tobacco cessation, lipid management, blood pressure control, diet, weight management, and diabetes screening. In addition, Dr. Rodriguez presented the model used by MD Anderson for forming a survivorship care team and clinic model. Following this, cancer survivor, Ms. Vicky Duck, discussed her real-life experience from the patient perspective after she developed late cardiovascular side effects in survivorship. She highlighted the importance of having a support team not only from her family but from her physicians who took extra time to listen and address her needs and concerns.

### Cardio-oncology rehabilitation program

The importance of cardio-oncology rehabilitation and cardiovascular fitness were presented by Dr. Susan Gilchrist. Cardiovascular reserve is not only dependent on cardiac and pulmonary function but also on vascular compliance and skeletal muscle function which are both affected by cancer itself and cancer therapeutics. Multiple insults from comorbidities and direct and indirect results of cancer therapy can be reversed or mitigated by exercise. Dr. Gilchrist presented algorithms she developed and recently published in Circulation regarding the use of Cardio- Oncology rehabilitation in cancer patients [5]. She then discussed strategies that can be used for starting cardio-oncology rehabilitation programs.

### Supportive/palliative care

Dr. Ali Haider of the Department of Supportive Care discussed the role of supportive/palliative care in the management of cardio- oncology patients across the continuum of care. His presentation included advance care planning, ethics of care, and when should of end-of life care be discussed in Cardio-Oncology patients. He also discussed how to care for patients in challenging situations, and how to integrate Supportive/palliative care in daily decision making. Dr. Haider emphasized the aim of palliative care is to clinically manage complex issues, provide psycho-social support to the patient and their family, to improve quality of remaining life, achieve the best possible death, and should be available from the point of diagnosis through to the end of life.

## Conclusion

Improved cancer survivorship and the increasing number of novel anti-cancer agents with potential cardiotoxic effects has led to the rapidly emerging development of cardio-oncology programs. Cancer patients with de novo and preexisting cardiovascular disease have complex needs and calls for a multidisciplinary approach for cardio-oncology care. The multidisciplinary cardio-oncology team includes cardiologists, oncologists, advanced practice providers (nurse practitioners, physician assistants), pharmacists, nurses, dieticians, social workers, and other allied health personnel involved in the management of these patients. However, Cardio-Oncology education is frequently absent in cardiology and oncology training programs. Moreover, education for multidisciplinary teams are lacking. The Cardio-Oncology Multidisciplinary Practice (COMP) 2020 conference was developed to provide education to the multidisciplinary cardio-oncology team members collectively, reduce the knowledge gaps and improve consistency in patient care. Studies have shown that multidisciplinary programs have been associated with enhanced patient well-being and improved clinical outcomes. More studies and emphasis on practical application of these programs are needed; future conferences will explore a combination of face-to-face and remote access to the sessions, allowing selective participation as well as full access without the need to be physically present, as has been the model for previous Cardio-Oncology sessions.

## Data Availability

Data sharing not applicable to this article as no datasets were generated, analyzed, or used/disclosed during the meeting described in this paper.
